# Enhancing EEG-Based Mental Stress State Recognition Using an Improved Hybrid Feature Selection Algorithm

**DOI:** 10.3390/s21248370

**Published:** 2021-12-15

**Authors:** Ala Hag, Dini Handayani, Maryam Altalhi, Thulasyammal Pillai, Teddy Mantoro, Mun Hou Kit, Fares Al-Shargie

**Affiliations:** 1 School of Computer Science & Engineering, Taylor’s University, Jalan Taylors, Subang Jaya 47500, Malaysia; alahag85@gmail.com (A.H.); thulasyammal.ramiahpillai@taylors.edu.my (T.P.); 2 School of Computer Science, Nusa Putra University, Jl. Raya Cibolang No.21, Sukabumi 43152, Indonesia; dini.odh@nusaputra.ac.id; 3 Department of Management Information System, College of Business Administration, Taif University, P.O. Box 11099, Taif 21944, Saudi Arabia; 4 Faculty of Engineering and Technology, Sampoerna University, Jakarta 12780, Indonesia; teddy.mantoro@sampoernauniversity.ac.id; 5 Department of Mechatronic and Biomedical Engineering, Universiti Tunku Abdul Rahman, Kajang 43000, Malaysia; munhk@utar.edu.my; 6 Department of Electrical Engineering, American University of Sharjah, Sharjah P.O. Box 26666, United Arab Emirates; fyahya@aus.edu

**Keywords:** brain–computer interface (BCI), electroencephalography (EEG), stress state recognition, feature selection, particle swarm optimization (PSO), mRMR, SVM, DEEP, SEED

## Abstract

In real-life applications, electroencephalogram (EEG) signals for mental stress recognition require a conventional wearable device. This, in turn, requires an efficient number of EEG channels and an optimal feature set. This study aims to identify an optimal feature subset that can discriminate mental stress states while enhancing the overall classification performance. We extracted multi-domain features within the time domain, frequency domain, time-frequency domain, and network connectivity features to form a prominent feature vector space for stress. We then proposed a hybrid feature selection (FS) method using minimum redundancy maximum relevance with particle swarm optimization and support vector machines (mRMR-PSO-SVM) to select the optimal feature subset. The performance of the proposed method is evaluated and verified using four datasets, namely EDMSS, DEAP, SEED, and EDPMSC. To further consolidate, the effectiveness of the proposed method is compared with that of the state-of-the-art metaheuristic methods. The proposed model significantly reduced the features vector space by an average of 70% compared with the state-of-the-art methods while significantly increasing overall detection performance.

## 1. Introduction

Mental stress is one of the apprising issues globally, which affects almost everyone. It is considered one of the major contributing causes to various serious health issues. To avoid its negative impact, scientists and psychologists have suggested detecting mental stress at an early stage before becoming chronic. Therefore, the most common method of evaluating mental stress is known as a subjective method that uses self-report questionnaires such as the perceived stress scale [1]. The issue of subjective methods is that they are inconvenient and require a lot of time for better assessments. Many people ignore regular evaluation until co-related health problems become apparent; thus, it is not applicable for real-life applications. However, objective methods assessment such as EEG is considered one of the promising tools for building real-life applications, helping individuals assess themselves without the need for experts’ involvement. However, building such an application needs an efficient method for EEG analysis, such as employing the most related channels and features to the mental state task. As a result, feature selection and channel selection methods play an essential role in enhancing the classification performance, reducing system complexity, and increasing diagnoses’ convertibility [2].

Feature selection (FS) is a crucial processing step in machine learning that leads to the development of efficient real-world applications. FS methods aim to identify the most relevant feature sets for a given task by removing irrelevant and redundant features. Hence, it reduces the high dimensionality space and prevents the incidence of the over-fitting problem caused by the curse of dimensionality [2,3]. In particular, FS approaches have demonstrated their significant impact on improving the overall classification performance of a given problem in terms of the quality of the extracted features and reducing the computational costs [4]. Therefore, the current state of the art efforts employs FS as an optimization technique to improve classification performance by selecting the best feature set [2]. Furthermore, FS has been effectively used to address a variety of classification issues in various domains, such as pattern recognition [5,6,7], data mining [2], and other domains where high dimensionality occurred.

Specifically, pattern recognition such as electroencephalography (EEG) is a vulnerable domain requiring the extraction of relevant patterns from high dimensional space. Current research studies employed EEG to acquire brain activities because it is reliable, affordable, portable, and provides high temporal resolution of the brain signals’ activities [8]. In multichannel EEG, several features extracted from the time domain, frequency domain, time-frequency domain, spatial domain, etc., contribute to form a high dimensional feature space in which one aims to recognize or assess several brain states such as seizure detection (epilepsy) [9], motor imaginary [10], depression [11], emotion detection [12,13], and mental stress recognition [14]. Thus, many feature extraction methods (in the time, frequency, and time-frequency domains) have been employed to extract meaningful information from an EEG signal associated with a mental task. Although researchers utilized a variety of feature extraction approaches, from frequency domain (beta, alpha, theta, gamma, and delta bands), time domain, or time-frequency domain, the majority of researchers have employed time-frequency features such as fast Fourier transform (FFT) [1,15] and wavelet transform (WT) [16,17] of (alpha, beta, gamma) because they yield more accurate results than time-domain features alone. Additionally, those frequencies have been quantified mathematically using various techniques, including relative power, absolute power, power ratio, spectral power, alpha asymmetry, and valance/arousal [18,19,20,21]. Researchers frequently combined two or more feature extraction approaches [1,22].

Recently, EEG signals have been used extensively in the field of emotion recognition, particularly in recognition of distress due to harmful influences on physical and mental health [1,23]. However, one of the significant challenges in building a successful model for stress detection is finding the most appropriate features. Due to this challenge, researchers employ several features extracted from the time domain, frequency domain, brain connectivity network or time-frequency network, and a combination of one or more methods to find their association with mental stress [1], despite significant efforts from community researchers in recent studies to enhance the classification accuracy of mental stress state recognition using EEG signals, few important studies utilized multi-feature domains and multi-channel EEG with the feature selection method. Yet, there is no solid conclusion of what is the optimal feature subset for stress recognition [5,6,24]. Consequently, current researchers acknowledge that multi-feature and multi-channel analyses are required to establish informative feature space in which a good interpreter can eventually produce effective alarms of the occurrence of mental state [6,12,15,24,25], allowing subjects to seek appropriate treatment at an early stage. The success in finding an optimal feature set for stress detection would be an essential step toward creating real-world applications that would provoke clinical or behavioural intervention if stress continued to worsen [26]. However, extracting EEG multi-features from different domains would result in high dimensionality that may contain irrelevant features that are not helpful for machine learning classification due to the enormous search space known as the “curse of dimensionality” [3]. Thus, FS becomes an essential pipeline to address these problems in EEG-specific domain analysis by selecting an optimal feature subset and reducing system complexity [3,27].

The feature selection approaches are often categorized into two main types: filter and wrapper methods. Some other selection approaches are discussed in various literature [3,28]. Filter methods measure the degree of the importance between each feature without the involvement of subsequent learning algorithms [29]. In contrast, wrapper methods rely on prediction models (e.g., SVM and KNN) to estimate the importance of features via classification algorithm evaluation [2]. In comparison, filter methods usually outperform wrapper methods in terms of computational speed because they use statistical measurements between features such as feature distance, information gain, and feature dependency; however, wrapper methods proved to be better at finding the importance of feature subsets that improve overall classification performance [29,30]. Nonetheless, these two feature selection approaches still suffer from some drawbacks, such as the feature selected subset can present a correlation between features (redundancy), be trapped in local optima, and may trigger a high computational cost [31,32]. Moreover, they tend to perform global searches to find the optimal features, yet it is impossible in most cases [33].

An efficient global search technique is needed to select a (near) optimal feature subset from the original feature set to address the above feature selection challenges. With global searchability, the swarm intelligence-based heuristic search methods have superior performance in obtaining optimal solutions [28] compared with filter and wrapper approaches. Metaheuristics are also superior to random searches because they can perform as a comprehensive search method (filter methods) in the worst-case scenario. Swarm intelligence-based (SI) heuristic search methods aim to investigate the behaviour of a group of agents in self-organized communities, such as ants, bees, moths, and birds [3]. Recently, several SI algorithms, such as the ant colony optimization (ACO) [34], bee optimization algorithm (BeOA) [35], moth-flame optimizer (MFO) [36], multi-verse optimizer (MVO) [37], butterfly optimization algorithm (BOA) [38], bat algorithm (BAT) [39], firefly algorithm (FFA) [3], grey wolf optimizer (GWO) [40], moth optimization algorithm (MOA) [36], whale optimization algorithm (WOA) [2], and particle swarm optimization (PSO) [2,41], have been successfully utilized to discover the optimal feature subset. However, despite the excellent findings, most of these algorithms have a poor convergence rate and are entrapped in local optima [38]. Therefore, researchers developed various hybrid algorithms using swarm intelligence models, which fused at least two approaches to improve each method’s performance and overcome challenges that occurred in search space [3,42].

PSO is a relatively recent optimization technique that is more computationally efficient than the existing metaheuristic methods. The method has been shown to be a valuable solution for optimization issues due to its effectiveness and ease of implementation. However, there are some significant issues with the conventional PSO when used for feature selection, such as lack of exploitation for particular problems [43]. Moreover, no feature selection method can handle all optimisation problems based on the “NO Free Lunch” (NFL) theorem. As a result, if one algorithm outperforms other algorithms in one specific domain problem, it may not exceed them in another. Furthermore, the standard PSO is suggested for the continuous optimization problem, which cannot be employed for feature selection issues with binary solution space [43].

Therefore, the abovementioned considerations motivate us to propose a new hybrid method to make it more suited for feature selection and effectively balance exploration and exploitation in the search process.

This work proposes a hybrid of minimum-redundancy maximum relevance and PSO (mRMR-PSO) feature selection methods. The proposed method aims to improve the exploitation of the PSO algorithm. The mRMR is utilized to enhance the exploitation of PSO as a building block of PSO. It ranks a set of features by applying Pearson’s correlation to minimize the redundancy among the subset of features while maximizing the features’ relevance using the f-test. The output of the selected optimal features is then classified using a support vector machine (SVM).

In summary, the main contributions of this work are given as follows:

1. Develop an experimental protocol to induce stress on participants while solving mental arithmetic tasks under time pressure and negative feedback.
2. Extract multi-domain features from multi-EEG channels and fuse them to form a large pool of feature vectors.
3. Propose a novel EEG feature selection method called mRMR-PSO-SVM to improve the search of local optimal and fit for binary feature selection.
4. Validate the proposed method by utilizing our dataset with another three public datasets of EEG on mental stress state and compare its performance with several metaheuristic algorithms.

The structure of the paper is organized as follows. Section 3 explains the datasets’ structures, preprocessing, and feature extraction and presents the proposed framework’s methodology. In Section 4, the results of the study are presented. Finally, the discussion and conclusion are provided in Section 5 and Section 6, respectively.

## 2. Experiment and Materials

In this study, our EEG dataset for mental stress state (EDMSS) and three other public datasets were utilized to validate the proposed method. The below subsections describe the details for each dataset.

### 2.1. Participants

A total of 22 healthy right-handed males (aged 26 ± 4 with a head size of 56 ± 2 cm) participated in this experiment. The experiment was conducted between 3:00 p.m. and 4:30 p.m. to avoid circadian rhythm influences on the alpha-amylase measurement. These individuals had no psychiatric, neurological, or psychotropic drug use history. The participants were placed in a comfortable room with good air conditioning and instructed to avoid moving their heads as much as possible throughout the experiment to prevent any environmental stress. All participants were informed about the study and signed consent forms before the trial began. The study protocol was designed following the declaration of Helsinki and was approved by the Institutional Review Board at the American University of Sharjah, 19-513/31/03/2020.

### 2.2. Stress Inducement Method

We induced stress by utilizing mental arithmetic tasks with time pressure and negative feedback as reported in our previous studies [16,26,44]. Then, we carried out the experimental protocol in four steps.

Step 1: The participants were given a brief introduction to their assigned tasks, and an alpha-amylase sample (S1) was collected as a baseline.

**Figure 1:**
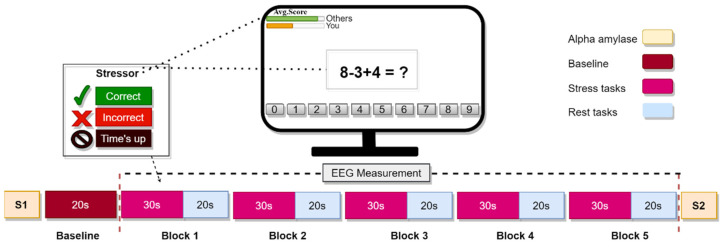
Experiment block design. A total of five blocks for the stress and rest tasks. In each block, arithmetic tasks were generated for 30 s followed by 20 s of rest. Alpha-amylase samples (S1–S2) were taken five minutes before the experiment began as a baseline and five minutes after the experiment ended.

Step 2 (training phase): Each participant practised the mental arithmetic (MA) task for five minutes to estimate how long it would take to answer each question. The MA task involved three single-digit integers (range from 0 to 9) and used two operators; plus (+) or minus (−) (for example, 7 − 3 + 1), see Figure 1. Each question’s response was displayed on a computer monitor in the order of ‘0’ to ‘9’, and the participant had to select the correct answer with a single left click on the mouse.

Step 3 (stress phase): Participants performed the same MA task under time pressure and negative emotional feedback. In this context, the allocated time to answer the MA task was reduced by 10% compared with the average time to answer each question during step 2. Participants who answered incorrectly or did not submit their answer within the allocated time received negative comments in the form of a notification display in the monitor such as “incorrect”, “time’s up”, or “correct” alongside the average performance of participants and the top excellent user.

Step 4 (rest phase): Participants were asked to look at a fixation cross on the computer with a black background to sustain their attention to the monitor. The entire experiment lasted about 15 min including the experimental setup. Meanwhile, the task presentation during the stress and rest states lasted five minutes. This experiment displayed the MA task in a block design following the fNIRS protocol [45,46].

The task’s block architecture is shown in Figure 1. Each block began with a 30 s MA stress task, followed by a 20 s rest task. The baseline was taken for a total of 20 s before starting the experiment. Immediately, at the end of the five blocks of the MA task, we collected the second sample of alpha-amylase (S2), as shown in Figure 1. To check if the participants were paying attention to the exercise, they calculated the accuracy with which they answered the questions. The average score was 95% accuracy in the training phase and 40% in the stress phase, as reported in the original published MIST paper [16].

### 2.3. Data Acquisition

**Figure 2:**
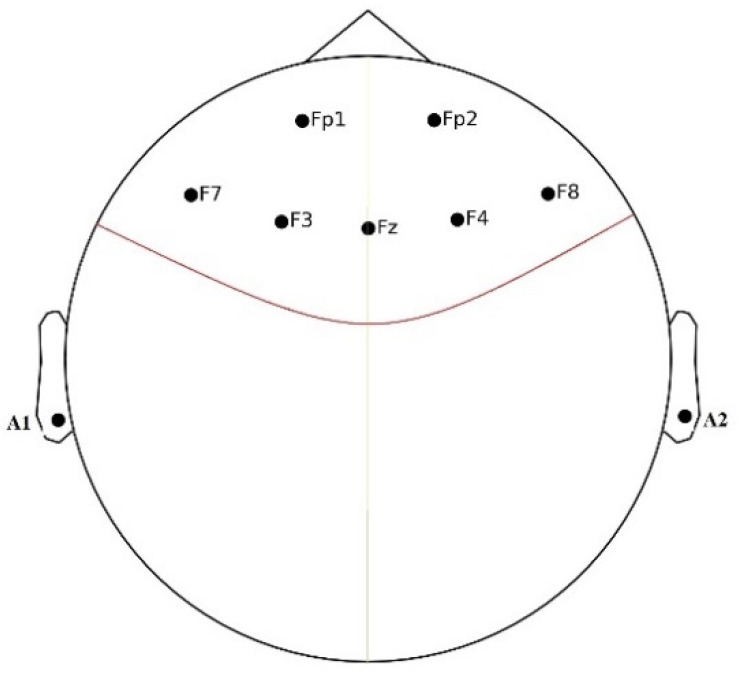
EEG electrodes’ placement on the scalp.

Brain activity was recorded using EEG signal from the prefrontal cortex using seven electrodes: FP1, FP2, F3, F4, Fz, F7, and F8, plus two reference electrodes, A1 and A2, placed on the earlobes as shown in Figure 2. The BrainMaster 24E system is an EEG machine with wet electrodes utilized in this study with a sampling rate of 256 Hz. However, we measure the cortisol activities using a hand-held monitor called COCORO meter (Nipro, Osaka, Japan).

### 2.4. Description of Public Datasets

Three publicly available datasets were also used in this study to validate the proposed method. The summary of the datasets’ contents data used in this study is shown in Table 1. The description for each of the three datasets are given below:

#### 2.4.1. DEAP Dataset

A Database for Emotion Analysis using Physiological Signals (DEAP) is a well-known publicly available dataset for emotion classification [47]. The DEAP dataset contains multiple physiological signals for the evaluation of emotions. Thirty-two healthy participants participated in the experiment. The EEG signals were acquired with 32 channels/electrodes while watching music videos. A total of 40 different music videos were used, each 60 s long for emotional stimulation. Then, the signals were downsampled to 128 Hz and preprocessed from artefacts and noise. The EEG signals were cleaned from EOG artefacts, de-noised using bandpass filters from 4–45 Hz, and data were averaged to a common reference. In the DEAP dataset, the emotional state was labelled based on arousal and valence of self-assessment manikins (SAM) [48].

The EEG signals were annotated based on the online self-assessment rating SAM scale provided by DEAP for valence and arousal. Based on Russell’s model for emotion representation, valence denotes the stimulus’s pleasantness on a negative to positive scale. In contrast, arousal refers to the intensity of emotion elicited by the stimulus, ranging from calm (or low) to excited (or high). Valence denotes the stimulus’s pleasantness on a negative to positive scale. In contrast, arousal refers to the intensity of emotion elicited by the stimulus, ranging from calm (or low) to excited (or high). A calm state is considered when arousal is low, and valence is high. Meanwhile, the stress state is induced by a low valence and a high level of arousal [49]. Therefore, in this dataset, the valence and arousal values were considered to annotate calm and stress tasks for each participant using Equations (1) and (2), derived from [24,50]:

(1) Calm=(arousal<4)∩(4<valence<6)  

(2) Stress=(arousal>5)∩(valence<3)  

By applying the rules of selecting stress and calm states from each participant, a result of 25 participants met the rules, and seven participants (with participant IDs: 3, 6, 7, 9, 17, 23, and 30) were excluded. Therefore, in our study, the rest of the DEAP analysis continued with the remaining data of 25 participants.

#### 2.4.2. SEED Dataset

SJTU Emotion EEG Dataset (SEED) is a public emotion dataset consisting of 15 subjects (seven males and eight females), aged 23.27 ± 2.37 (mean ± std)) participating in the emotion-inducing experiment. Each subject was required to watch 15 selected film clips with positive, neutral, and negative stimuli to induce a corresponding emotional state with a duration of 4 min long of each film clip. Three sessions of data were collected, and each session comprised 15 trials/movies. The data were recorded using 62 EEG channels with a 1000 sampling rate from each participant. The placement of 62 EEG channels was determined according to the international 10–20 system. Then, the data were downsampled to 200 Hz to reduce computing complexity. A bandpass filter from 0–75 Hz was applied. In this paper, we only used the positive and negative labels/classes to compare 2 class problems of the other emotional state datasets. In summary, 45 files (3 experiments for each participant) were used with the data shape (trail, channels, and samples data) and label file of the 15 trails. More details about the dataset can be found in [51].

#### 2.4.3. EDPMSC Dataset

The EEG Dataset for Classification of Perceived Mental Stress (EDPMSC) is a publicly available dataset that contains the EEG physiological signals of 28 participants (13 men and 15 women, ages 18–40) [25]. The EDPMSC contains data collected at 256 sampling rates from four Muse headband dry EEG channels. These electrodes are AF7, AF8, TP9, and TP10 and are placed on the scalp as references using Fz. The PSS questionnaire was used to assess a subject’s level of stress over the preceding 30 days, which was then used to categorize EEG signals as stress or not stress. Each participant underwent three experiments. The first experiment was termed the pre-active phase, during which EEG data were collected for three minutes while sitting in a relaxed position in a quiet room with open eyes. The second phase involved recording EEG data during a presentation (activity phase) in front of people. Finally, there was a three-minute post-activity phase during which EEG data were collected in the recording room. If the PSS score was more significant than or equal to twenty, the subject was classified as stressed; if it was less than twenty, the subject was classified as non-stressed. The author of the dataset compared the pre-active and post-active phases and concluded that the pre-active phase is more accurate at identifying stress. As a result, we employ the precative phase in this study to develop our proposed model. The raw EEG data were preprocessed using a bandpass filter of a finite impulse response (FIR) filter with a bandwidth of 0.5 Hz to 35 Hz. A high bandpass filter with a cutoff frequency of 0.5 Hz was chosen to eliminate slow drifts. Additionally, the low bandpass filter of 35 Hz was used to eliminate line noise at 50 Hz and store data for the delta, theta, alpha, and beta frequency bands.

## 3. Methodology

**Figure 3:**
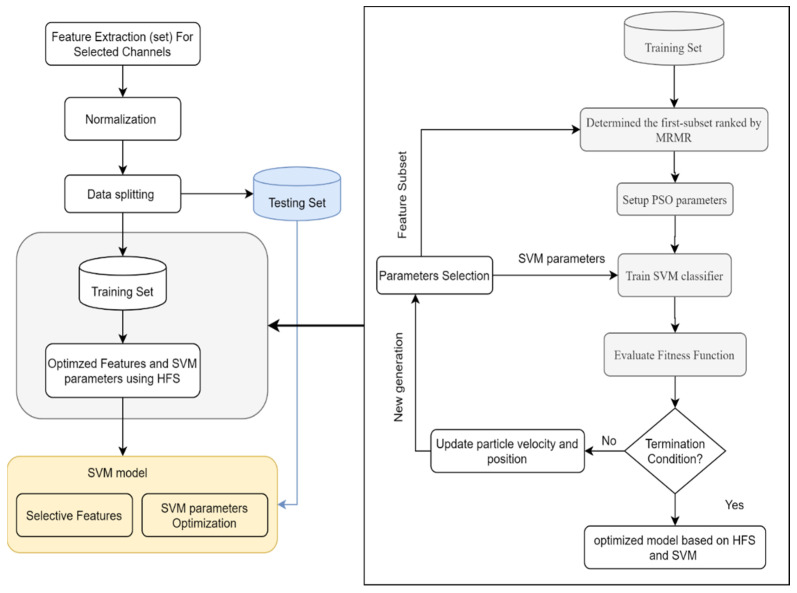
The flowchart of the proposed feature selection method mRMR-PSO-SVM.

A hybrid dominant feature selection method is developed in this study to enhance the classification performance of EEG mental stress recognition. A block diagram of the proposed method is shown in Figure 3. Multi-domain features are extracted from the frequency domain, time-frequency domain, and connectivity features. The optimal subset selected features are used to classify mental stress state using optimized SVM. The method’s implementation phases are outlined below.

- Dataset preprocessing
- Multi-domain features are extracted from multi-EEG channels and combined to form a large feature vector.
- Feature selection based on the proposed mRMR-PSO method identifies discriminative features.
- Classification parameters of SVM were optimized using PSO.
- The proposed model was validated with three different public datasets.

### 3.1. Data Preprocessing

The preprocessing of EEG signal was implemented using Python and an external package called MNE. Raw EEG signals were preprocessed using a bandpass filter. Finite impulse response (FIR) filters between 0.5 Hz to 35 Hz were used to remove DC artefacts and line noise (50/60 Hz). All EEG channels were subjected to the common average reference. Fast, independent component analysis was used to eliminate the eye blink generated by electrooculogram (EOG), eye movements, and muscular artefacts. After that, the clean signals were employed for the rest of the research.

### 3.2. Feature Extraction

We extracted multi-domain features that best discriminate mental stress levels and enhance classification accuracy in this work. Features from the time domain, frequency domain, time-frequency domain, and connectivity features were extracted. The connectivity features were estimated by utilizing a phase-locking value. Features from the time domain were based on Hjorth parameters of activity mobility and complexity, peak to peak amplitude, line length, kurtosis, and skewness. Frequency domain features were based on the relative power of theta (4–8 Hz), alpha (8–12 Hz), sigma (12–15 Hz), low beta (15–20 Hz), and high beta (20–30 Hz). Likewise, time-frequency domain features were based on spectral entropy (PSD, Welch) [12] and Katz fractal dimension [1,35]. These features have been employed in several EEG studies and shown to be useful [1]. Table 2 shows the summary of all features used in this study. These features were then normalized using feature-based z-score normalization to avoid large-scale weighting. 

### 3.3. Feature Selection Using mRMR-PSO

The proposed mRMR-PSO method consists of minimum redundancy maximum relevance (mRMR) [57] and a PSO algorithm. The proposed method aims to select the more informative feature subsets related to mental stress from the high dimensional space of the EEG signal to improve the accuracy performance of the SVM classifier by ranking the relative and informative features. We first utilized the filter method of mRMR to generate a short feature pool and PSO wrapper to get the least redundant feature set and optimized SVM parameters for better accuracy. The sections below describe the details of each method.

#### 3.3.1. Minimum-Redundancy Maximum Relevance (mRMR)

The mRMR is a filter-based method that was first proposed by Ding and Peng (2005) [58] as a solution for feature selection problems and has been shown to be computationally fast. The mRMR is used to rank a subset of features by minimizing the redundancy between the subset of features while maximizing the relevance of the feature to the target.

The proposed method employs mRMR to minimize the search space of the local optima of the original feature by ranking a subset of important features. The mRMR algorithm is based on a relevance measurement using an F-score, while the redundancy measurement is based on Pearson’s correlation among the features set [59]. This process minimizes the selection of redundant features, which results in minimizing the risk of overfitting at the PSO phase and solving the issue of global search space.

The maximum relevance (*RL*) is computed using the F-statistic *F(X_i,_ y*) between feature and target class as the equation:

(3) $$maxRL_{f},RL_{f}=\frac{1}{\left|S\right|}\sum_{X_{i}\in S}F(X_{i},y)$$

where *S =* $\left\{X_{1},X_{2},X_{3},\ldots ,X_{n}\right\}$ is the set of features, *y* is the target class (e.g., stress/not, stress), and *|S*| is the size of the feature set.

The minimum redundancy (*RD*) among features is computed using Pearson’s correlation between a pair of features as shown in Equation (4):

(4) $$\min RD_{\rho },RD_{\rho }=\frac{1}{{\left|S\right|}^{2}}\sum_{X_{i}\in S}\rho (X_{i},X_{j})$$

The full join formula of the mRMR selection schema to rank the feature set is calculated using the *F*-test correlation quotient $f^{FCQ}$ as the equation:

(5) $$f^{FCQ}(X_{i})=\underset{X_{i}\in \Omega_{S}}{\max}\left\{\frac{F(X_{i},y)}{\frac{1}{\left|S\right|}\sum_{X_{j}\in S}\left|\rho (X_{i},X_{j})\right|}\right\}$$

where $\rho (X_{i},X_{j})$ is the Pearson’s correlation between a pair of features, *F(X_i_,y)* is F-statistic, *X_i_* (*i* ∈ {1, 2, ..., m}) is feature importance based on the mRMR criterion, m is total features, |*S*| is the size of the feature set, and *y* refers to the target (class/label). In summary, at each stage of the mRMR feature selection process, the features with the highest feature important score will be added to the subset $f^{FCQ}(X_{i})$ selected feature ranks. The $f^{FCQ}$ mRMR feature selection results in achieving more coverage balance in the solution space as well as contributing significant improvements to classification performance.

#### 3.3.2. PSO Algorithm

Particle swarm optimization (PSO) was proposed by Kennedy and Eberhart (1995) for optimization problems [43,60,61,62]. PSO is a swarm intelligence metaheuristic technique motivated by social behaviour such as fish schooling and birds searching for food. PSO is based on the concept of birds exchanging information with one another. When birds seek food at random, they have no idea where to look. Like the evolutionary and genetic algorithms, PSO searches on a population (called swarm) of individuals (called particles), updated from iteration to iteration. PSO discovers the optimal solution by allowing each particle to change its searching direction based on two factors: the best of all features (gbest) and its own best previous experience (pbest).

The status of each particle is characterized based on its position (global optima) and velocity (distance: local optima). If the position of each particle found its best position, then the information would be delivered to other particles. The particles’ velocity and position were updated over iteration to search for pbest and gbest for optimal solution p as equations.

(6) $$\begin{array}{ll} V_{id}^{t+1}= & w\cdot V_{id}^{t}+c_{1}\cdot r_{1}(pbest_{id}^{t}-X_{id}^{t})\\ & +c_{2}\cdot r_{2}(gbest_{id}^{t}-X_{id}^{t}) \end{array}$$

(7) $$X_{id}^{t+1}=X_{id}^{t}+V_{id}^{t+1},d=\{1,2,\ldots ,D\}$$

where *t* denotes evolutionary generation, $V_{id}^{t}$ denotes a particle’s velocity i on dimension *d*, $X_{id}^{t}$ denotes a particle’s position i on dimension *d*, ($c_{1}$,$c_{2}$) denotes social learning factors of personal best (pbest) and global best (gbest), respectively, and ($r_{1}$,$r_{2}$) are random numbers of uniformly distributed U (0,1). The w refers to the weight used to balance global exploration and local exploitation.

#### 3.3.3. Proposed Hybrid Method: mRMR-PSO-SVM

This section proposes a hybrid method mRMR-PSO-SVM proposed for mental stress classification, as shown in Figure 3. The mRMR-PSO-SVM algorithm aims to select the optimal feature set from the reduced set of $f^{FCQ}$ mRMR while optimizing the classification performance by estimating the optimal values of SVM parameters (C, γ) simultaneously.

In our approach, three main phases are considered for better optimization: initialization, feature selection, and classification and evaluation.

*In the initialization phase:* it is proved that the high number of particles (P) increases the computational complexity of the optimization process. In contrast, the small search space of P results in poor optimal solutions [62]. Therefore, we select 200 and 50 for a total number of generations (t) and a total number of particles, respectively. Likewise, ($c_{1}$,$c_{2}$) and w values influence the convergence of the optimization process. If set too high, the particle velocity becomes too fast, and the optimum solution cannot be obtained. Thus, we set ($c_{1}$ and $c_{2}$) to 2 and w was set to mean the mutual information of the subset selection $f^{FCQ}$.

*In the feature selection phase:*

The mRMR algorithm is developed as described in Section B.1 to rank the most important datasets’ features, resulting in the highest classification performance with the SVM classifier. The mRMR-PSO evaluates each selection of ranked feature subset and SVM parameters according to a fitness function, the classification F-measure of SVM.

*Classification and evaluation phase:*

Support vector machines (SVM) have been widely used in different applications, including EEG-based applications [63,64]. The classification procedure is a part of wrapper feature selection methods to evaluate and validate the model. In our method, we optimize SVM parameters (C, γ) using PSO and evaluate the subset performance using the activation function of F-measure as Equations (8)–(10):

(8) $$precision=\frac{TP}{TP+FP}$$

(9) $$recall=\frac{TP}{TP+FN}$$

(10) $$F-measure=2\cdot \frac{precision\cdot recall}{precision+recall}$$

where *TP* refers to the total number of true positives, false positives (*FP*), true negatives (*TN*), and false-negatives (*FN*). Accuracy is also used as an overall measure for classification, which is:

(11) $$accuracy=\frac{TP+TN}{TP+TN+FP+FN}$$

Additionally, SVM has different kernel functions such as linear, polynomial, and radial basis functions (RBF). This study utilises RBF to obtain optimal solutions because it is widely used when dealing with multi-dimensional space. Moreover, the number of parameters that need to be optimized are few compared with other kernels such as polynomial. RBF has two parameters: C and γ. Parameter C denotes the cost of the penalty. The choice of value for C influences the classification outcome. At the same time, parameter γ has a much greater influence on classification outcomes than C because its value affects the partitioning outcome in the feature space [65]. The primary aim is to select a suitable kernel function and its kernel parameter(s) because the kernel defines the feature space in which the training sets will be classified. In this context, the values of RBF parameters need to be optimized for the optimal use of the SVM along with the feature selection.

In summary, the main basic procedure for the proposed algorithm (mRMR-PSO-SVM) is presented as follows:

Step 1: Use the mRMR method to rank the features of the training sets from the highest best feature to the lowest using Equation (5). -

Step 2: Initialize PSO parameters (populations, number of particles, learning parameters ($c_{1}$, and $c_{2}$), the inertia weight (w), and the generate velocity and position of each particle).

Step 3: Train the selected subset of the features from step 1 using RBF-SVM.

Step 4: Evaluate the selected features with PSO and SVM parameters using the fitness function of F-measure as shown in Equation (10) -

Step 5: Update parameters of PSO (velocity and position) and SVM parameters (C, γ) until the termination criteria are met.

Step 6: Termination condition: recursively use steps 3, 4, and 5 for refining the model fitness until the criteria of termination are satisfied (e.g., number of generations or accuracy fulfilled).

Step 7: Classify mental stress from testing data using the generated optimal model (optimal selected model and optimized parameters).

## 4. Result

This section presents the statistical results of the mental stress experiment induced by MAT and assessed by EEG and alpha-amylase. We report the mental stress state classification based on optimal feature set, selected from multi-domain features, of network connectivity features, time domain, frequency domain, and time-frequency domains, using the proposed method mRMR-PSO-SVM.

### 4.1. Statistical Analysis

**Figure 4:**
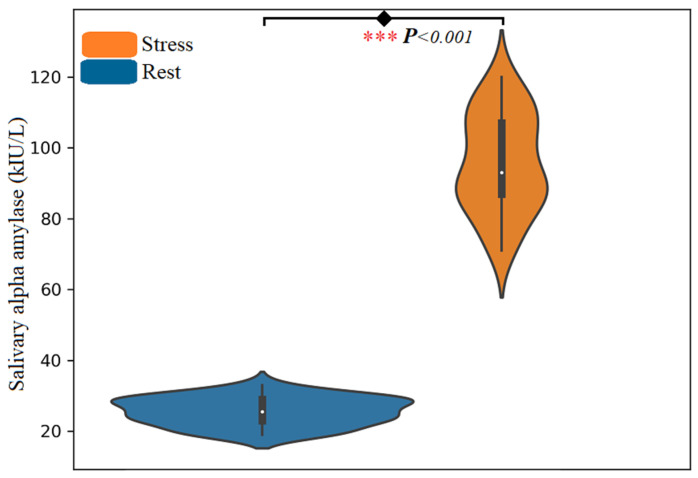
The average score of salivary alpha-amylase level responses for stress and rest tasks. Two measurement samples (5 min before (baseline) and 5 min after the last stress task). The “***” marks indicate the task is significant with *p* < 0.001.

In our EDMSS experiment, the stress tasks were induced using a mental arithmetic task with negative feedback and time pressure. The salivary alpha-amylase (SAA) was used to assess and validate mental stress during EEG acquisition and plays as a biomarker for EEG annotation. The mean scores acquired from 22 participants using the SAA are shown in Figure 4.

Overall, the reported SAA level among participants’ scores were (µ, σ) = (24.45 ± 4.44 (kIU/L)) before stress inducement and (µ, σ) = (93.64 ± 13.99 (kIU/L)) after stress inducements. Participants with an SAA score of more than 60 (kIU/L) were classified as being in a stress state, whereas those with a score of less than 30 (kIU/L) were classified as being in the rest group. The t-test was applied on SAA to verify the effects of stress inducements on rest and stress states. The difference between the mental states is considered significantly different if the *p*-value is less than 0.05. The results revealed a significant difference between the two states with *p* < 0.001. The approach of stress inducement using mental arithmetic tasks used in this experiment is similar to that used by other researchers [1].

### 4.2. Performance Analysis of Feature Selection and Multi-Domain Features

We evaluated the performance of our methodology using EDMSS and further validated it using three public EEG datasets. The datasets DEAP, SEED, and EDPMSC were utilized here for mental stress recognition. The datasets DEAP, SEED, and EDPMSC were utilized here for mental stress recognition. A summary of the datasets is provided in Table 1, which shows the number of EEG channels used from each dataset. In the DEAP and SEED datasets, eight channels were selected, mostly from the prefrontal and frontal regions of the brain; seven EEG channels were selected from EDMSS, and 4 EEG channels were selected from EDPMSC.

The multi-domain features were extracted from each dataset and used as input vectors after normalizing them using column-base z-score normalization. The features of multi-domains are combined to derive a high-dimensional feature vector. Table 1 shows the number of EEG channels used from each dataset. In DEAP and SEED datasets, eight channels were selected, mostly from the prefrontal and frontal regions of the brain; seven EEG channels were selected from EDMSS, and 4 EEG channels were selected from EDPMSC.

The multi-domain features were extracted from each dataset and used as input vectors after normalizing them using column-base z-score normalization. The features of multi-domains are combined to derive a high-dimensional feature vector.

Table 2 summarises the proposed multi-domain features that provide the domain name, feature description, total number features, and feature formula. A total of 161 multi-domain features were extracted from the seven EEG channels of EDMSS, 188 features for the datasets containing eight channels (DEAP, SEED), and only 86 features were extracted from the EDPMSC dataset containing 4 EEG channels.

**Figure 5:**
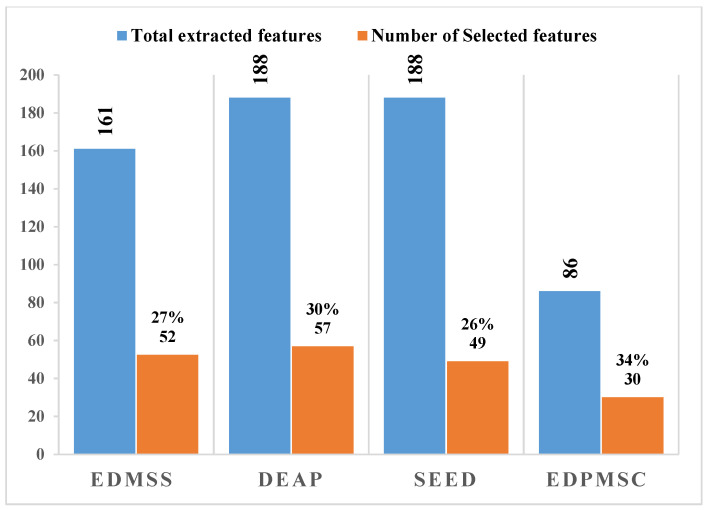
A total number of multi-domain features were selected using mRMR-PSO-SVM.

**Figure 6:**
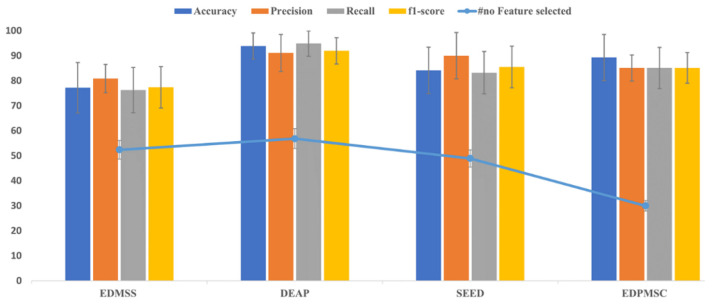
The evaluation performance of the proposed mRMR-PSO-SVM on different EEG datasets for mental stress detection.

Figure 5 and Figure 6 represent the results of mRMR-PSO-SVM in selecting the optimal feature subset per dataset. The figures show that mRMR-PSO-SVM can significantly reduce a large number of feature vector spaces while achieving a high classification performance. Figure 6 shows the classification performance results and the number of selected features obtained by our proposed algorithm on different datasets. The highest average classification performance on EDMS was 77.23%, 80.87%, 76.30%, and 77.41% for accuracy, precision, recall, and f1-score, respectively, with an average of 52 optimal selected features. In the DEAP dataset, the proposed algorithm achieved an average performance of 93.88%, 91.11%, 94.91 %, and 91.99% for accuracy, precision, recall, and f1-score, respectively, with an average of 56 selected features. Additionally, for the SEED dataset, the achieved results were 84.17 for accuracy, 90% precision, 83.23% recall, and 85.54 f1-score using 49 optimal selected features. In the EDPMSC dataset, the results obtained, based on dependent subject analysis since each subject was labelled as a stressed subject or not, were 89.31%, 85.11%, 85.11%, and 85.11% for accuracy, precision, recall, and f1-score, respectively, with only 45 selected features.

To verify the performance of the proposed model mRMR-PSO-SVM with the DEAP dataset, we evaluated and compared the results with other SI algorithms, as shown in Table 3 and Table 4. The average performance of the model optimized by mRMR-PSO-SVM is 93.88%, 91.11%, 94.91 %, and 91.99% for accuracy, precision, recall, and f1-score, respectively, with an average of 56 selected features based on the selected ranges of the two dimensions of valence and arousal. For example, previous studies [24,50] reported that stress state could be considered if the arousal is higher than scale five and valence less than scale 3, as shown in Equation (2). Yet, in general, these scales are subjective assessment methods that are totally dependent on the subject feedback and could lead to an increase in the error rates of assessment [1]. Therefore, we performed a Friedman test to analyze whether significant differences exist among the performance of the small changes of arousal and valence ranges (example arousal = 4.9, 5, or 4.8). We found that only four subjects with ids (22, 25, 27, and 28) had these scores in the used DEAP dataset and found significant differences according to the Friedman test, *F_r_* = 8, *p*-value = 0.018316. The significant differences in the obtained results confirm the sensitivity of our proposed objective EEG method (EDMS). Therefore, the small limits of valence and arousal and mean threshold of each mental state should be further investigated and validated using different objective assessment methods such as cortisol level.

From Figure 5, the proposed algorithm reduced the total number of features to less than 70% from all datasets’ original feature vector space while increasing the prediction accuracy or maintaining it.

The mRMR-PSO-SVM approach preserved just around 30% of the features on all four datasets while improving prediction accuracy. The findings indicate that the proposed mRMR-PSO-SVM method can efficiently remove redundant or irrelevant features, resulting in better classification performance.

To evaluate the effectiveness of the proposed mRMR-PSO-SVM method, we compared it with other states of the art swarm intelligence metaheuristic algorithms, namely, BAT, FFA, GWO, MFO, MVO, PSO, and WOA [66]. The performance of each feature selection method was assessed using three parameters, namely, classification accuracy, execution time, and the number of selected features utilized for mental stress classification. For that, a split mechanism was used to train and test feature selection methods to obtain the classification accuracy with 80% for training and 20% for testing. 

Table 3 shows the average accuracy, selected features, and execution time for each swarm optimizer algorithm with EDMSS, DEAP, SEED, and EDPMSC datasets. In DEAP, the proposed algorithm achieved the highest accuracy of 93.878% using 57 selected features, while the highest among the compared algorithms was MVO with 88.877% accuracy and 86 features. Meanwhile, in the SEED dataset, the proposed method obtained an accuracy of 84.167% with only 49 selected features, higher than the best competitive optimizer FFA that performed 74.815% of accuracy using 90 features with the same dataset. Additionally, mRMR-PSO-SVM obtained 77.22% and 88.301% accuracy with 52 and 30 selected features for EDMSS and EDPMSC, respectively. However, in EDPMSC, the WOA showed a slight increase in accuracy with 1.026% using 36 selected features.

## 5. Discussion

This present study investigated EEG mental stress recognition using a hybrid feature selection method. In addition, the study developed an experimental protocol to induce stress on the participants while doing mental arithmetic tasks under time constraints. The findings of the experimental work showed that the proposed stress task significantly increased the salivary cortisol level of all subjects with *p* < 0.01. Furthermore, the EEG features extracted from the collected EDMSS dataset achieved a high classification accuracy of 77.2% using 55 features from the seven active electrodes. This confirms the visibility of using EEG as a biomarker of stress classification at a minimum of 20 s.

The proposed hybrid feature selection method of mRMR-PSO-SVM aims to select an optimal number of features that increase or maintain the overall classification performance by enhancing the exploration and exploitation of search space.

Various time domain, frequency domain, and time-frequency domain features have been proposed in previous studies for mental stress detection [1,13,23]. However, little research utilizes connectivity features. Our study utilizes the most important features from multiple domains, seeking better informative features for stress detection. As a result, a fusion of multi-domain features showed a promising result in different fields as there could be multi-way interactions among features [14,67]. The drawback of multi-domain features is that they are vulnerable to redundant and unrelated features. Therefore, the proposed mRMR-PSO-SVM method aims to select the discriminative features from high dimensional space. The optimal selected features using mRMR-PSO are used to train SVM with the RBF kernel, then evaluate the classification performance with test data. In each dataset, an independent subject test was conducted.

Swarm intelligence (SI) has quickly evolved in recent years and relatively provide an efficient solution for tackling NP-hard computational problems, such as high-dimensionality features [3,68]. FS is also seen as an optimization issue, with methods aiming to select a subset of important features that balances accuracy while minimizing the number of features required. The effective SI-based feature selection strategies should use a range of search algorithms to create a robust and adaptable method, with a better objective of balancing exploration and exploitation capabilities and providing a faster convergence rate. Table 3 shows that our PSO variation has a larger exploration than exploitation ratio, allowing it to learn the feature space more effectively. However, it cannot always find the optimum solution based on the results. Depending on the dataset, the exploration and exploitation variables of PSO should be adjusted independently. This is consistent with another study, which found that typical PSOs have unbalanced exploration and exploitation and could be improved by adjusting its parameters [68]. Among the SI algorithms, the bat algorithm (BAT), grey wolf optimizer (GWO), moth optimization algorithm (MVO), and whale optimization algorithm (WOA) performed the best in all factors: enhancing accuracy and lowering feature number while maintaining an acceptable execution time. The firefly algorithm (FFA) has a longer excursion time when searching for a solution other than SI algorithms. This could be due to a problem with its strategy process. Only one firefly moves randomly according to chaotic search, limiting its global searchability, which researchers addressed by proposing several modifications to improve global searchability [69]. According to [68], GWO is superior to PSO and BAT algorithms. This is because BAT takes longer to discover the optimal solution, whilst PSO requires extra parameter tweaking to achieve the best result. However, based on the results, it is clear that MOA outperforms other SI algorithms and deserves greater attention and research. 

This shows that the proposed mRMR-PSO-SVM outperforms the other algorithms used in this study regarding the accuracy and selected features. The significant advantage in finding the optimum number of features is justified since the proposed mRMR-PSO-SVM employs the strength of mRMR and PSO mechanisms, which enhances the possibility of selecting weak solutions and decreases the probability of trapping in local optima. This method allows the algorithm to fully explore parts of the feature space before using PSO to improve these regions.

To address feature selection problems, this approach uses the strengths of a global search algorithm, which is efficient in both exploration (local search) and exploitation (global search). Finding fewer optimum features means that the algorithm has successfully removed irrelevant/redundant features from the feature vector space of the dataset. However, excursion time shows slightly better than FFA in the EDMSS and SEED datasets while it takes more time than other optimizers. This could be due to the hybrid interactions between the filter method (mRMR) and wrapper method (PSO).

It is worth mentioning that the proposed mRMR-PSO method outperforms the conventional PSO algorithm in classification accuracy and most of state of the art meta-heuristic algorithms, namely, BAT, FFA, GWO, MFO, MVO, PSO, and WOA [66]. As a result, the mRMR-PSO is a promising technique for detecting significant factors while removing redundant and irrelevant data. Compared with the original PSO algorithm with the same approach, we can conclude that the proposed mRMR-PSO method offers accurate classification performance with the fewest number of selected features in all datasets. Thus, the mRMR-PSO algorithm provides a viable solution to feature selection and mental stress classification problems.

To further highlight the efficacy of the proposed hybrid feature selection method, the mRMR-PSO-SVM showed superior performance compared with the state of the art feature selection methods in terms of classification performance, based on the number of selected features as listed in Table 4. From Table 3 andTable 4, we can highlight that our proposed method outperforms other proposed methods in terms of accuracy and the number of selected features. This could be due to many factors tackled in our model, such as fusion of multi-domain features, improving PSO exploitation, and optimizing the SVM parameters.

One feature selection method was chosen and assessed in this experiment from swarm intelligence-based algorithms to compare various approaches to selecting a feature set. All of the approaches studied in this section were chosen from wrapper-based methods to ensure a fair evaluation and comparison [3]. Due to the fundamental differences between filter-based and wrapper-based feature selection approaches, in which filter-based models often have lower computational complexity, and wrapper-based models typically have higher accuracy, these methods cannot be compared. To provide a fair comparison, the experiments in this section were evaluated and compared using two techniques; the first technique examined our proposed approach with similar state of the art methods of SI algorithms as shown in Table 3, while the second technique compared filter-based methods proposed by the previous state of the art studies which employ it with the same public datasets as shown in Table 4.

For example, [50] proposed a wrapper method of genetic algorithm (GA) with KNN with DEAP dataset and achieved an accuracy of 71.76%. Similarly, [24] employed a wrapper method of Boruta-KNN to select optimal features and achieved an accuracy of 73.38%. However, our proposed hybrid method achieved an accuracy of 93.87% due to the ability to select the most discriminative features from feature vector space.

**Table 4:** Comparison with previous studies on related publicly available datasets for mental stress detection.

#Ref.	Dataset	FS-Classifier	Total Feature Vector/ Selected Features	No. Channels	Accuracy
[50]	DEAP	GA- KNN	673/not mentioned	32	71.76%
[24]	DEAP	Boruta-KNN	608/288	32	73.38%
[25]	EDPMSC	Wrapper FS- (MLP, SVM)	90/18	4	89.30% MLP, 67.85% SVM for pre-active phase
[23]	DEAP	2-D AlexNet-CNN 3-D AlexNet-CNN	5 PSD bands converted to image	32	84.77%, 86.12%
[70]	SEED, DEAP	DWT-BODF (SVM, KNN)	225 × 30 SEED 576 × 40 DEAP	62 SEED 32 DEAP	93.8% SVM (SEED) 77.4% SVM (DEAP)

It is worth emphasizing that our proposed model focused on EEG-based mental stress only. However, it could be further utilized with other datasets from different domains.

To summarize, the study’s findings are quite promising. However, there is still much potential for additional research in the field of EEG-based mental stress classifications. First, we conducted our study using a fixed time window length; however, experimenting with alternative window lengths can aid model generalization. Additionally, it is worth exploring data augmentation via sliding windows as this technique is increasingly being utilized in combination with deep learning on EEG [23]. Second, a more in-depth examination of other connective network features should be explored since they give detailed information about channel interconnections. Our method was based on established signal noise removal techniques [1]. However, alternative denoising techniques such as signal smoothing should be considered.

Additionally, prior research has demonstrated that utilizing feature extraction with feature selection approaches and neural networks results in high accuracy in EEG-based models. As a result, future studies may potentially explore neural networks and deep learning approaches. Finally, our research utilized four datasets that use both subjective and objective assessment methods. However, subjective methods such as the SAM questionnaire depend on a set of ranges within the valence and arousal scale. These self-assessments of manikin should be supported with objective methods such as cortisol level or behavioral responses.

## 6. Conclusions

In this paper, a hybrid feature selection method, mRMR-PSO-SVM, was proposed to select the most informative features related to the mental stress task. By ranking important features as a subset of the original feature set, the mRMR was used to reduce the search space of the local optima of the original feature set. Following that, PSO evaluates each ranked feature subset selection and optimizes SVM parameters according to a fitness function. The mRMR-PSO-SVM was evaluated using four datasets and compared with existing methods based on selected features and classification performance. According to the experimental results obtained on various datasets, the proposed method outperformed other feature selection methods regarding the accuracy and the selected channels. Even though the proposed method yielded promising results, future research could further validate the methods with different domain datasets and use neural networks and deep learning approaches to better evaluate the quality of selected features and their effects on computation time.

## Figures and Tables

**Table 1:** A summary description of the datasets used in this study.

Dataset	Stimuli (Stressor)	Stress Labelling	Total EEG Channels	Selected Channels	No. Participants/ Total Experiments	Frequency Rate (Hz)	Classes
DEAP	Music video	SAM	32	AF3’, ‘FC5’, ‘F8’, ‘Fp1’, ‘AF4’, ‘P7’, ‘Fp2’, ‘F7	32/32	128	Stress/ calm
SEED	Emotional video	Questionnaire	62	‘AF3’, ‘FC5’, ‘F8’, ‘Fp1’, ‘AF4’, ‘P7’, ‘Fp2’, ‘F7’	15/45	200	Negative/positive
EDPMSC	History	PSS	4	‘TP9’, ‘AF7’, ‘AF8’,’TP10’	28/84	256	Stress/ not stress
Our	MA, negative feedback and time pressure	Saliva cortisol	7	‘Fp1’, ‘Fp2’, ‘F7’, ‘F3’, ‘Fz’, ‘F4’, ‘F8’	22/22	256	Stress/ rest

**Table 2:** Summary of multi-domain feature extraction methods employed in the selected datasets.

Domain	Feature Name	Description	No. Features	Formula
Connectivity	Phase Locking Value [52]	It is a proportion of phase difference between signals over different trials above or below the 0 degree	$\frac{n(n-1)}{2}$	$PLV_{ij}=\left|\frac{1}{T}\sum_{i=1}^{N}e^{i(\phi_{t}^{a}-\phi_{j}^{a})}\right|$
Time	Hjorth parameters of activity mobility, and complexity [28]	Activity is the variance of the signal on-time.	1	Activity=var(y(i))
		Mobility represents the proportion of standard deviation of the window signal in the time domain.	1	$Mobility=\sqrt{\frac{{\mathrm{var}}^{\prime }(y(i))}{Activity}}$
		Complexity represents how the shape of a signal is similar to a pure sine wave.	1	$Complexity=\sqrt{\frac{Mobility(\frac{dy(t)}{dt})}{Mobility(y(t))}}$
	Peak to peak amplitude	Represents the peak time of EEG signal between the various windows.	1	$\begin{array}{l} PTP=pk_{high}-pk_{low} \end{array}$
	Line length [28,53]	Named a curve length, which indicates the total vertical length of the signal.	1	$\begin{array}{l} L(n)=\sum_{i=1}^{N-1}x[i]-x[i-1] \end{array}$
	Kurtosis [54,55]	Shows the sharpness of EEG signals’ peaks.	1	$\begin{array}{l} Kurtosis \end{array}=\frac{\frac{1}{t}\sum_{t=1}^{T}{\left(x(t)-µ\right)}^{3}}{\sigma 4}$
	Skewness [17]	Represents the asymmetry of an EEG signal.	1	$Skewness=\frac{\frac{1}{t}\sum_{t=1}^{T}{\left(x(t)-µ\right)}^{3}}{\sigma 3}$
Frequency	Relative powers of [18]: Theta (4–8 Hz) Alpha (8–12 Hz) Sigma (12–15 Hz) Low beta (15–20 Hz)A high beta (20–30 Hz).	Relative power represents the average absolute power of the given band intervals.	5	$RP=\frac{power(band)}{power(All\_bands)}*100$
Time-Frequency	Spectral entropy (PSD, Welch) [12,56]	Measures the distribution of signal power over frequency.	1	$SE=-\sum_{f=4}^{F=45}\bar{PSD}(F)log(\bar{PSD}(F))$
	Katz fractal dimension [35]	Represents the maximum distance between the first point and any other point of the signal’s time window.	1	$D=\frac{log_{10}(n)}{log_{10}(\frac{L}{d})+log_{10}(n)}$

**Table 3:** The average values of the statistical parameters of classifiers using the subject independent test.

Algorithm	Execution Time	Accuracy	#No Selected Features	Execution Time	Accuracy	#No Selected Features
	EDMSS DATASET			EDPMSC DATASET		
BAT	4.315	67.624	75	15.378	87.703	44
FFA	19.615	65.172	79	19.285	87.935	36
GWO	9.234	67.664	74	15.001	87.703	55
MFO	4.336	67.267	85	16.586	88.167	55
MVO	4.135	67.631	80	14.620	88.863	45
PSO	5.530	65.289	108	15.923	84.919	55
WOA	5.773	64.224	72	15.195	89.327	36
Proposed	11.719	77.222	52	60.700	88.301	30
	DEAP DATASET			SEED DATASET		
BAT	10.328	88.229	80	2.946	68.889	86
FFA	41.391	88.079	87	14.852	74.815	90
GWO	21.013	87.515	83	6.939	71.111	84
MFO	46.348	88.182	97	2.865	70.370	85
MVO	10.695	88.877	86	2.869	70.370	85
PSO	13.682	88.276	121	4.027	66.667	122
WOA	14.482	88.697	79	4.236	68.148	79
Proposed	53.768	93.878	57	9.346	84.167	49

## Data Availability

Raw EEG data of EDMSS’ dataset can be obtained by writing a formal email to Fares Al-Shargie.

## References

[1] Katmah R., Al-Shargie F., Tariq U., Babiloni F., Al-Mughairbi F., Al-Nashash H. (2021). A Review on Mental Stress Assessment Methods Using EEG Signals. Sensors.

[2] Hussien A.G., Oliva D., Houssein E.H., Juan A.A., Yu X. (2020). Binary whale optimization algorithm for dimensionality reduction. Mathematics.

[3] Rostami M., Berahmand K., Nasiri E., Forouzandeh S. (2021). Review of swarm intelligence-based feature selection methods. Eng. Appl. Artif. Intell..

[4] Kang X., Handayani D.O.D., Chong P.P., Acharya U.R. (2020). Profiling of pornography addiction among children using EEG signals: A systematic literature review. Comput. Biol. Med..

[5] Pei Z., Wang H., Bezerianos A., Li J. (2021). EEG-Based Multiclass Workload Identification Using Feature Fusion and Selection. IEEE Trans. Instrum. Meas..

[6] Tuncer T., Dogan S., Subasi A. (2021). EEG-based driving fatigue detection using multilevel feature extraction and iterative hybrid feature selection. Biomed. Signal Process. Control.

[7] Angga Yuwono H., Kusuma Wijaya S., Prajitno P. (2020). Feature selection with Lasso for classification of ischemic strokes based on EEG signals. J. Phys. Conf. Ser..

[8] Molla M.K.I., Al Shiam A., Islam M.R., Tanaka T., Tanaka T., Tanaka T. (2020). Discriminative Feature Selection-Based Motor Imagery Classification Using EEG Signal. IEEE Access.

[9] Tzimourta K.D., Astrakas L.G., Gianni A.M., Tzallas A.T., Giannakeas N., Paliokas I., Tsalikakis D.G., Tsipouras M.G. (2018). Evaluation of window size in classification of epileptic short-term EEG signals using a Brain Computer Interface software. Eng. Technol. Appl. Sci. Res..

[10] Sun Z., Huang Z., Duan F., Liu Y. (2020). A Novel Multimodal Approach for Hybrid Brain–Computer Interface. IEEE Access.

[11] Movahed R.A., Jahromi G.P., Shahyad S., Meftahi G.H. (2021). A major depressive disorder classification framework based on EEG signals using statistical, spectral, wavelet, functional connectivity, and nonlinear analysis. J. Neurosci. Methods.

[12] Yin Y., Zheng X., Hu B., Zhang Y., Cui X. (2021). EEG emotion recognition using fusion model of graph convolutional neural networks and LSTM. Appl. Soft Comput..

[13] Halim Z., Rehan M. (2020). On identification of driving-induced stress using electroencephalogram signals: A framework based on wearable safety-critical scheme and machine learning. Inf. Fusion.

[14] Hag A., Handayani D., Pillai T., Mantoro T., Kit M.H., Al-Shargie F. (2021). EEG Mental Stress Assessment Using Hybrid Multi-Domain Feature Sets of Functional Connectivity Network and Time-Frequency Features. Sensors.

[15] Subhani A.R., Mumtaz W., Saad M.N.B.M., Kamel N., Malik A.S. (2017). Machine Learning Framework for the Detection of Mental Stress at Multiple Levels. IEEE Access.

[16] Al-shargie F., Tang T.B., Badruddin N., Kiguchi M. (2018). Towards multilevel mental stress assessment using SVM with ECOC: An EEG approach. Med. Biol. Eng. Comput..

[17] Hosseini S.A., Khalilzadeh M.A., Naghibi-Sistani M.B., Homam S.M. (2015). Emotional stress recognition using a new fusion link between electroencephalogram and peripheral signals. Iran. J. Neurol..

[18] Asif A., Majid M., Anwar S.M. (2019). Human stress classification using EEG signals in response to music tracks. Comput. Biol. Med..

[19] Bachmann P., Schächinger H., Naumann E., Schilling T.M., Zhang X., Larra M.F. (2018). Emotional stress regulation: The role of relative frontal alpha asymmetry in shaping the stress response. Biol. Psychol..

[20] Cheema A., Singh M. (2019). Psychological stress detection using phonocardiography signal: An empirical mode decomposition approach. Biomed. Signal Process. Control.

[21] Minguillon J., Lopez-Gordo M.A., Pelayo F. (2016). Stress Assessment by Prefrontal Relative Gamma. Front. Comput. Neurosci..

[22] Gedam S., Paul S. (2021). A Review on Mental Stress Detection Using Wearable Sensors and Machine Learning Techniques. IEEE Access.

[23] Martínez-Rodrigo A., García-Martínez B., Huerta Á., Alcaraz R. (2021). Detection of Negative Stress through Spectral Features of Electroencephalographic Recordings and a Convolutional Neural Network. Sensors.

[24] Hasan M.J., Kim J.M. (2019). A Hybrid Feature Pool-Based Emotional Stress State Detection Algorithm Using EEG Signals. Brain Sci..

[25] Arsalan A., Majid M., Butt A.R., Anwar S.M. (2019). Classification of Perceived Mental Stress Using A Commercially Available EEG Headband. IEEE J. Biomed. Heal. Inform..

[26] Al-Shargie F., Tang T.B., Kiguchi M. (2017). Stress Assessment Based on Decision Fusion of EEG and fNIRS Signals. IEEE Access.

[27] Cvetkovic D., Übeyli E.D., Cosic I. (2008). Wavelet transform feature extraction from human PPG, ECG, and EEG signal responses to ELF PEMF exposures: A pilot study. Digit. Signal Process. A Rev. J..

[28] Boonyakitanont P., Lek-uthai A., Chomtho K., Songsiri J. (2020). A review of feature extraction and performance evaluation in epileptic seizure detection using EEG. Biomed. Signal Process. Control.

[29] Toradmalle D., Muthukuru J., Sathyanarayana B. (2019). Hybrid Feature Selection Method based on Particle Swarm Optimization and Adaptive local Search Method. Int. J. Electr. Comput. Eng..

[30] Mafarja M.M., Mirjalili S. (2017). Hybrid Whale Optimization Algorithm with simulated annealing for feature selection. Neurocomputing.

[31] Ghanem W.A.H.M., Jantan A. (2016). Novel multi-objective artificial bee colony optimization for wrapper based feature selection in intruction detectoin. Int. J. Adv. Soft Comput. Appl..

[32] Xue B., Zhang M., Browne W.N. (2013). Particle Swarm Optimization for Feature Selection in Classification: A Multi-Objective Approach. IEEE Trans. Cybern..

[33] Venkatesh B., Anuradha J. (2019). A review of Feature Selection and its methods. Cybern. Inf. Technol..

[34] Ma W., Zhou X., Zhu H., Li L., Jiao L. (2021). A two-stage hybrid ant colony optimization for high-dimensional feature selection. Pattern Recognit..

[35] Garro B.A., Salazar-Varas R., Vazquez R.A. EEG Channel Selection using Fractal Dimension and Artificial Bee Colony Algorithm. Proceedings of the IEEE Symposium Series on Computational Intelligence (SSCI).

[36] Mirjalili S. (2015). Moth-flame optimization algorithm: A novel nature-inspired heuristic paradigm. Knowl. Based Syst..

[37] Jangir P., Parmar S.A., Trivedi I.N., Bhesdadiya R.H. (2017). A novel hybrid Particle Swarm Optimizer with multi verse optimizer for global numerical optimization and Optimal Reactive Power Dispatch problem. Eng. Sci. Technol. Int. J..

[38] Sadeghian Z., Akbari E., Nematzadeh H. (2021). A hybrid feature selection method based on information theory and binary butterfly optimization algorithm. Eng. Appl. Artif. Intell..

[39] Bablani A., Edla D.R., Tripathi D., Dodia S., Chintala S. (2019). A Synergistic Concealed Information Test with Novel Approach for EEG Channel Selection and SVM Parameter Optimization. IEEE Trans. Inf. Forensics Secur..

[40] Naserbegi A., Aghaie M., Zolfaghari A. (2020). Implementation of Grey Wolf Optimization (GWO) algorithm to multi-objective loading pattern optimization of a PWR reactor. Ann. Nucl. Energy.

[41] Ahmed M.A., Qi D., Alshemmary E.N. (2020). Effective hybrid method for the detection and rejection of electrooculogram (EOG) and power line noise artefacts from electroencephalogram (EEG) mixtures. IEEE Access.

[42] Agrawal P., Abutarboush H.F., Ganesh T., Mohamed A.W. (2021). Metaheuristic algorithms on feature selection: A survey of one decade of research (2009–2019). IEEE Access.

[43] Ji B., Lu X., Sun G., Zhang W., Li J., Xiao Y. (2020). Bio-Inspired Feature Selection: An Improved Binary Particle Swarm Optimization Approach. IEEE Access.

[44] Al-Shargie F., Tang T.B., Badruddin N., Kiguchi M. Simultaneous measurement of EEG-fNIRS in classifying and localizing brain activation to mental stress. Proceedings of the IEEE 2015 International Conference on Signal and Image Processing Applications ICSIPA.

[45] Al-Shargie F., Tang T.B., Kiguchi M. (2017). Assessment of mental stress effects on prefrontal cortical activities using canonical correlation analysis: An fNIRS-EEG study. Biomed. Opt. Express.

[46] Al-Shargie F., Kiguchi M., Badruddin N., Dass S.C., Hani A.F.M., Tang T.B. (2016). Mental stress assessment using simultaneous measurement of EEG and fNIRS. Biomed. Opt. Express.

[47] Koelstra S., Muhl C., Soleymani M., Lee J., Yazdani A., Ebrahimi T., Pun T., Nijholt A., Patras I. (2012). DEAP: A Database for Emotion Analysis; Using Physiological Signals. IEEE Trans. Affect. Comput..

[48] Özerdem M.S., Polat H. (2017). Emotion recognition based on EEG features in movie clips with channel selection. Brain Inform..

[49] Al Zoubi O., Awad M., Kasabov N.K. (2018). Anytime multipurpose emotion recognition from EEG data using a Liquid State Machine based framework. Artif. Intell. Med..

[50] Shon D., Im K., Park J.H., Lim D.S., Jang B., Kim J.M. (2018). Emotional Stress State Detection Using Genetic Algorithm-Based Feature Selection on EEG Signals. Int. J. Environ. Res. Public Health.

[51] Zheng W.L., Zhu J.Y., Lu B.L. (2019). Identifying stable patterns over time for emotion recognition from eeg. IEEE Trans. Affect. Comput..

[52] Al-Shargie F., Tariq U., Alex M., Mir H., Al-Nashash H. (2019). Emotion Recognition Based on Fusion of Local Cortical Activations and Dynamic Functional Networks Connectivity: An EEG Study. IEEE Access.

[53] Esteller R., Echauz J., Tcheng T., Litt B., Pless B. (2001). Line length: An efficient feature for seizure onset detection. Annu. Int. Conf. IEEE Eng. Med. Biol..

[54] Alimardani F., Cho J.H., Boostani R., Hwang H.J. (2018). Classification of bipolar disorder and schizophrenia using steady-state visual evoked potential based features. IEEE Access.

[55] Arsalan A., Majid M. (2021). Human stress classification during public speaking using physiological signals. Comput. Biol. Med..

[56] Li Y., Hu B., Zheng X., Li X. (2019). EEG-Based Mild Depressive Detection Using Differential Evolution. IEEE Access.

[57] Direito B., Duarte J., Teixeira C., Schelter B., Le Van Quyen M., Schulze-Bonhage A., Sales F., Dourado A. (2011). Feature selection in high dimensional EEG features spaces for epileptic seizure prediction. IFAC Proc. Vol..

[58] Peng H., Long F., Ding C. (2005). Feature selection based on mutual information criteria of max-dependency, max-relevance, and min-redundancy. IEEE Trans. Pattern Anal. Mach. Intell..

[59] Zhao Z., Anand R., Wang M. Maximum Relevance and Minimum Redundancy Feature Selection Methods for a Marketing Machine Learning Platform. Proceedings of the 2019 IEEE International Conference on Data Science and Advanced Analytics (DSAA).

[60] Mehmood R.M., Lee H.J. Emotion recognition from EEG brain signals based on particle swarm optimization and genetic search. Proceedings of the IEEE International Conference on Multimedia and Expo Workshops (ICMEW).

[61] Wu S.L., Liu Y.T., Hsieh T.Y., Lin Y.Y., Chen C.Y., Chuang C.H., Lin C.T. (2017). Fuzzy Integral with Particle Swarm Optimization for a Motor-Imagery-Based Brain-Computer Interface. IEEE Trans. Fuzzy Syst..

[62] Yang S.T., Der Lee J., Chang T.C., Huang C.H., Wang J.J., Hsu W.C., Chan H.L., Wai Y.Y., Li K.Y. (2013). Discrimination between Alzheimer’s disease and mild cognitive impairment using SOM and PSO-SVM. Comput. Math. Methods Med..

[63] Chen G., Chen J. (2015). A novel wrapper method for feature selection and its applications. Neurocomputing.

[64] Li X., Hu B., Sun S., Cai H. (2016). EEG-based mild depressive detection using feature selection methods and classifiers. Comput. Methods Programs Biomed..

[65] Lin S.W., Ying K.C., Chen S.C., Lee Z.J. (2008). Particle swarm optimization for parameter determination and feature selection of support vector machines. Expert Syst. Appl..

[66] Khurma R.A., Aljarah I., Sharieh A., Mirjalili S. (2020). EvoloPy-FS: An Open-Source Nature-Inspired Optimization Framework in Python for Feature Selection. Evolutionary Machine Learning Techniques.

[67] Tong L., Zhao J., Fu W. Emotion Recognition and Channel Selection Based on EEG Signal. Proceedings of the International Conference on Intelligent Computation Technology and Automation, ICICTA.

[68] Kicska G., Kiss A. (2021). Comparing Swarm Intelligence Algorithms for Dimension Reduction in Machine Learning. Big Data Cogn. Comput..

[69] Wang J., Zhang M., Song H., Cheng Z., Chang T., Bi Y., Sun K. (2019). Improvement and Application of Hybrid Firefly Algorithm. IEEE Access.

[70] Asghar M.A., Khan M.J., Fawad, Amin Y., Rizwan M., Rahman M., Badnava S., Mirjavadi S.S. (2019). EEG-based multi-modal emotion recognition using bag of deep features: An optimal feature selection approach. Sensors.

